# The Public’s views of mental health in pregnant and postpartum women: a population-based study

**DOI:** 10.1186/1471-2393-14-84

**Published:** 2014-02-24

**Authors:** Dawn E Kingston, Sheila Mcdonald, Marie-Paule Austin, Kathy Hegadoren, Gerri Lasiuk, Suzanne Tough

**Affiliations:** 1 5-258 Edmonton Clinic Health Academy, Faculty of Nursing, University of Alberta, 11405-87th Avenue, Edmonton, AB T6C 1C9, Canada; 2 Department of Pediatrics and Community Health Sciences, University of Calgary, Faculty of Medicine, 3280 Hospital Dr. NW, Calgary, AB T2N 4Z6, Canada; 3 University of New South Wales (AU), High St., Kensington NSW 2052, Australia

**Keywords:** Pregnancy, Postpartum, Mental health, Public views, Depression, Anxiety

## Abstract

**Background:**

We used population-based data to determine the public’s views of prenatal and postnatal mental health and to identify predictors of those views.

**Methods:**

A computer-assisted telephone survey was conducted by the Population Health Laboratory (University of Alberta) with a random sample of participants from the province of Alberta, Canada. Respondents were eligible to participate if they were: 1) ≥18 years; and 2) contacted by direct dialing. Questions were drawn from the Perinatal Depression Monitor, an Australian population-based survey on perinatal mental health; additional questions were developed and tested to reflect the Canadian context. Descriptive and multivariable regression analyses were conducted.

**Results:**

Among the 1207 respondents, 74.7% had post-secondary education, 16.3% were in childbearing years, and over half (57.4%) reported knowing a woman who had experienced postpartum depression. Significantly more respondents had high levels of knowledge of postnatal (87.4%) than prenatal (70.5%) mental health (p < .01). Only 26.6% of respondents accurately identified that prenatal anxiety/depression could negatively impact child development. Personal knowledge of a woman with postpartum depression was a significant predictor of prenatal and postnatal mental health knowledge.

**Conclusions:**

While the public’s knowledge of postnatal mental health is high, knowledge regarding prenatal mental health and its influence on child development is limited. Strategies for improving perinatal mental health literacy should target these knowledge deficits.

## Background

At a prevalence of 13 to 25%, stress, depression, and anxiety are the most common complications experienced by women during the prenatal and postnatal periods [1,2]. Poor mental health during the perinatal period is identified as a major public health concern and “the biggest unaddressed health challenge of our age” [3]. Without treatment, over one-third of women with prenatal mental health problems will experience unremitting symptoms that persist into their child’s preschool years [4]. However, despite frequent contact with healthcare professionals during pregnancy and post-delivery, the vast majority of women do not seek help for symptoms of stress, depression, or anxiety, or voluntarily disclose their symptoms [5,6].

Women cite significant deterrents to disclosure and help-seeking during the perinatal period [5-8]. While some barriers that women describe are clearly system-related (e.g., not obtaining a referral in a timely manner) [7], many reflect deeply held views regarding mental health problems in the prenatal and postnatal periods, including the expectation of being constantly happy to be pregnant or have a new infant, the association of mental health problems with maternal incompetency and being a failure as a mother, the fear that others would think less of them if they admitted to experiencing symptoms, the need to be a ‘super-mother’, the concern that their infant would be apprehended, and the belief that symptoms would improve spontaneously [8-10].

Views regarding mental health and healthcare are poignant determinants of patient behavior with regard to the access, uptake, and adherence to mental health treatment. Among pregnant and postpartum women, negative or inaccurate views of mental health are impediments to early detection and treatment, in that they are associated with delay in seeking mental healthcare [5] and reduced disclosure of mental health problems [6]. Understanding views towards mental health is also foundational to delivering effective intervention. Evidence rooted in the field of implementation science suggests that effective implementation of interventions must incorporate knowledge of the context that influences the impact of the intervention and its transference into clinical care - with patient views being a central component of the context [11,12]. Thus, effective and sustainable intervention utilizes patient views to optimize its impact and utilization [11].

While the public’s views surrounding mental health have been well studied with respect to general anxiety and depression, very little research has explored its views related to perinatal mental health. Three key observations highlight the importance of the investigation of the public’s views of perinatal mental health:

1) Only one study to date has explored the public’s views of perinatal mental health [13]; however, it is unclear whether these Australian findings are generalizable to other contexts in that they reflect the public’s awareness of perinatal mental health within a healthcare system that supports the National Perinatal Depression Initiative [14,15] and espouses highly visible, definitive perinatal mental health awareness strategies (http://www.beyondblue.org.au/);

2) Only a few, small studies have explored the views of mental health and healthcare among pregnant and postpartum women and their care providers [6,7,16-18]. Findings of these studies suggest that the most prominent attitudes toward mental health differ from studies conducted in the general population, and warrant a separate investigation. For example, surveys of public attitudes of mental health in the general population have identified social distance (e.g., not wanting to employ or befriend individuals with mental health problems) and stereotyping (e.g., as dangerous, to be blamed, unpredictable) as prominent concerns [19,20]. In the perinatal literature, attitudes toward mental health are uniquely embedded in concerns related to pregnancy or transitioning to motherhood, such as the belief that women with perinatal depression or anxiety are incompetent or bad mothers, and the guilt that originates from not feeling happy or being able to cope [6,7]; and

 3) The views of childbearing couples are highly sensitive to societal values and opinions, with family and friends representing dominant influences on their behaviors and decisions [5,8].

As such, understanding the public’s views regarding depression and anxiety in the prenatal and postnatal periods provides a valuable foundation for gaining insight into decision-making, help-seeking, and healthcare utilization patterns of childbearing families.

The purpose of this study was to use a large, population-based sample to describe the public’s views of perinatal mental health on topics related to knowledge of pre-conception, prenatal, and postnatal mental health and their influence on maternal and child outcomes, and to identify factors associated with perinatal mental health knowledge.

## Methods

### Overview

The Alberta Survey-B is an annual, population-based survey conducted by the Population Research Laboratory (PRL) at the University of Alberta (Alberta, Canada). Each year, different sponsors submit questions of interest. In 2012, the Alberta Centre for Child, Family and Community Research sponsored 15 items designed to gather information on views about perinatal mental health. Questions addressed topics related to knowledge of the effects of anxiety and depression occurring during pre-conception, pregnancy, and postpartum periods; help-seeking for prenatal and postpartum anxiety and depression; and screening and treatment for pregnant and postpartum women with anxiety or depression. Questions were drawn from the Australian *Perinatal Depression Monitor,* a 26-item survey designed to measure population-based awareness, attitudes, and knowledge regarding prenatal and postnatal mental health [13]; additional questions were developed and tested for this survey to address the Canadian context. The latter were evaluated for face and content validity by 15 researchers, clinicians, and policy-makers with expertise in perinatal mental health. Participants selected responses to items related to perinatal mental health on a 5-point Likert scale ranging from *strongly disagree* to *strongly agree*. All questions were pre-tested by the PRL on 20 households to check wording, response categories, question order, interviewer instructions, and length of interview [21].

Random-digit dialing of several provincial telephone banks generated a random sample of households from rural and urban areas and ensured that the sampling frame included households with and without telephone directory listings [21]. Respondents were eligible to participate if they were: 1) ≥18 years; and 2) contacted by direct dialing within the province. Following identification as an affiliate of the Population Health Laboratory and introduction to the study, agreement to participate in the telephone-based survey constituted consent. The study protocol was approved by the Research Ethics Board at the University of Alberta.

Data were collected between June 5, 2012 and June 27, 2012 by trained PRL interviewers using standardized, computer-assisted telephone interviews. Interviews were conducted in English and each lasted a maximum of 30 minutes. A random sample of 10% of the respondents were followed-up by PRL supervisors to verify accuracy of data collection.

### Outcomes

The main outcomes were knowledge of: 1) the influence of past history of anxiety and depression; 2) prenatal mental health and its adverse sequelae; and 3) postnatal mental health and its adverse consequences. For each outcome, the dichotomized definition of ‘high’ and ‘low’ knowledge was based on an examination of the distribution of the variable. Knowledge of the influence of past history was considered high if respondents answered *strongly* or *somewhat agree* to the question, “Women who have had anxiety or depression in the past (before they became pregnant) are more likely to experience anxiety or depression when they are pregnant”, and low if they responded *strongly* or *somewhat disagree*, or *neither agree nor disagree*. High level of prenatal mental health knowledge was defined by a response of *strongly* or *somewhat agree* to “Women who have had anxiety or depression in the past (before they became pregnant) are more likely to experience anxiety or depression when they are pregnant” and/or “Women who have anxiety or depression during pregnancy are more likely to experience postpartum depression”, while those with low prenatal knowledge answered *strongly* or *somewhat disagree* to either or both questions. High postnatal knowledge was defined by a response of *strongly* or *somewhat agree* to at least one of three questions, including, “Women who have postpartum depression find it more difficult to respond to their baby’s cues” and/or “Women who have postpartum depression find it more difficult to respond to the needs of their partner and other children” and/or “Partners of women who have postpartum depression are also at risk for depression”, whereas those with low postnatal knowledge responded *strongly* or *somewhat disagree* to one or more of these questions.

### Analysis

Descriptive data (N, %) were generated for each variable and outcome. The Chi-square test was used to determine differences between selected categorical variables. Unadjusted odds ratios (UORs) and 95% confidence intervals (CIs) were calculated for the outcomes of knowledge of prenatal and postnatal mental health. Variables associated with the outcomes at a level of p < .10 in unadjusted analyses were entered into the final multivariable models after assessing for the presence of multicollinearity. Two multivariable models were constructed with all variables entered simultaneously to generate adjusted odds ratios (AORs) and 95% CIs with p < .05 defining statistically significant factors. Excluded variables were entered one at a time to assess the robustness of the final models. The analysis was conducted using SPSS (Version 21.0.0).

## Results

### The sample

Of the total eligible participants (n = 10,563), 3029 refused, 29 interviews were incomplete, 102 interviews involved language problems, and 6196 were unavailable or not contacted (estimated eligible). In total, 1207 completed interviews were conducted with 603 females and 604 males. Approximately two-thirds of the sample lived in urban regions with one-third in rural areas. Respondents were most commonly 45–64 years of age (45.8%), with 16.3% being in childbearing years (18–34 years) (Table 1). Two-thirds of households had no children living in the household (66.3%). The majority of respondents were married or living common-law (68.6%), had completed post-secondary education (74.7%), were born in Canada (81.8%) and were Caucasian (85.4%) (Table 1). Over half of the respondents reported knowing a woman who had experienced postpartum anxiety or depression (57.4%).

**Table 1:** Description of respondents of the 2012 Alberta-B survey (Alberta, Canada) (N = 1207)

**Variable**	**n (%)**
Sex*	
Male	603 (50.0)
Female	604 (50.0)
Employment	
Unemployed	467 (38.7)
Employed	739 (61.3)
Children (age < 18) in household	
No	798 (66.3)
Yes	406 (33.7)
Age (years)	
18-24	53 (4.5)
25-34	139 (11.8)
35-44	211 (17.9)
45-54	269 (22.9)
55-64	269 (22.9)
65+	235 (20.0)
Marital status	
Single/widowed/divorced	377 (31.4)
Married/common-law	824 (68.6)
Education (highest level completed)	
Less than high school	96 (7.9)
High school completed	209 (17.4)
Post-secondary	898 (74.7)
Born in Canada	
No	220 (18.2)
Yes	987 (81.8)
Born in Alberta	
No	403 (40.9)
Yes	584 (59.1)
Ethnicity	
Non-Caucasian	186 (14.6)
Caucasian	1023 (85.4)
Income	
<$40,000	129 (13.7)
≥$40,000	815 (86.3)
Residence*	
Rural/other	401 (33.3)
Urban (planned)	804 (66.7)
Personal experience	
No	503 (42.6)
Yes	678 (57.4)

*Distribution planned a priori. Not all variables total N = 1207 due to missing data.

### Knowledge of influence of past history of anxiety and depression

Just over half of respondents strongly agreed/agreed that women with histories of anxiety or depression were more likely to experience anxiety or depression in pregnancy (57.2%) with 32.2% reporting not knowing (15.7%) or neither agreeing nor disagreeing (16.5%) (Table 2). A similar proportion strongly agreed/agreed that prior episodes of anxiety or depression in pregnancy increased women’s risk for postpartum depression (PPD) (60.9%). With respect to both of these questions, only one-quarter of respondents *strongly* agreed that a history of mental health problems increased women’s risk for poor prenatal and postnatal mental health (Table 2).

**Table 2:** Prevalence of outcomes (n, %) among respondents to the 2012 Alberta-B survey (Alberta, Canada) (N = 1207)

**Variable**	**n (%)**
Women who have had anxiety or depression in the past (before they became pregnant) are more likely to experience anxiety or depression when they are pregnant	
Strongly disagree	44 (3.6)
Disagree	83 (6.9)
Neither agree nor disagree	199 (16.5)
Agree	366 (30.3)
Strongly agree	325 (26.9)
No response/don’t know	190 (15.7)
Women who have anxiety or depression during pregnancy are more likely to experience postpartum depression	
Strongly disagree	33 (2.7)
Disagree	61 (5.1)
Neither agree nor disagree	203 (16.8)
Agree	423 (35.0)
Strongly agree	312 (25.9)
No response/don’t know	175 (14.5)
Women who have postpartum depression find it more difficult to respond to their baby’s cues	
Strongly disagree	28 (2.3)
Disagree	60 (5.0)
Neither agree nor disagree	150 (12.4)
Agree	411 (34.0)
Strongly agree	418 (34.6)
No response/don’t know	140 (11.6)
Women who have postpartum depression find it more difficult to respond to the needs of their partner and other children	
Strongly disagree	12 (1.0)
Disagree	26 (2.1)
Neither agree nor disagree	98 (8.2)
Agree	482 (39.9)
Strongly agree	482 (39.9)
No response/don’t know	107 (8.9)
Partners of women who have postpartum depression are also at risk for depression	
Strongly disagree	105 (8.7)
Disagree	160 (13.2)
Neither agree nor disagree	232 (19.3)
Agree	390 (32.3)
Strongly agree	155 (12.8)
No response/don’t know	164 (13.6)
Knowledge of prenatal mental health	
High	831 (70.5)
Low	347 (29.5)
Knowledge of postnatal mental health	
High	1028 (87.4)
Low	148 (12.6)

Not all variables total N = 1207 due to missing data.

### Knowledge of prenatal mental health

Over half of respondents agreed that women with anxiety or depression during pregnancy were more likely to experience PPD (60.9%) (Table 1). Only one-quarter of respondents (26.6%) agreed that children whose mothers were depressed or anxious during pregnancy were more likely to experience slower development; however, more than 40% of respondents reported that they did not know or were unsure. In the final multivariable model, participants who were older and did not know a woman who had experienced PPD were less likely to have high levels of knowledge of prenatal mental health (Table 3). Individuals who were not born in Canada were more likely to be knowledgeable than Canadian-born participants, as were those with a high level of knowledge of postnatal mental health.

**Table 3:** Unadjusted (UOR) and Adjusted Odds Ratios (AOR) of factors associated with knowledge of prenatal mental health in the 2012 Alberta-B survey (Alberta, Canada) (N = 1207)

**Variable**	**High level of prenatal mental health knowledge**	**Low level of prenatal mental health knowledge**	**UOR**	**95% CI**	**AOR**	**95% CI**
	**N (%)**	**N (%)**				
Sex						
Male	390 (67.2)	190 (32.8)	.73*	.57-.94	.81	.62-1.06
Female	441 (73.7)	157 (26.3)	1.00			
Employment						
Unemployed	314 (69.3)	139 (30.7)	.91	.70-1.17		
Employed	517 (71.4)	207 (28.6)	1.00			
Children (age < 18) in household						
No	543 (70.2)	231 (29.8)	.96	.73-1.25		
Yes	285 (71.7)	116 (28.9)	1.00			
Age (in childbearing years: 18–35)						
No	666 (69.2)	297 (30.8)	.54*	.36-.79	.52	.35-.78
Yes	151 (80.7)	36 (29.3)	1.00			
Marital status						
Single/widowed/divorced	267 (73.6)	96 (26.4)	1.23	.94-1.63		
Married/common-law	561 (69.3)	249 (30.7)	1.00			
Education						
No post-secondary education	212 (71.9)	83 (28.1)	1.08	.81-1.45		
At least some post-secondary education	617 (70.3)	261 (29.7)	1.00			
Born in Canada						
No	162 (75.7)	52 (24.3)	1.37*	.98-1.93	1.80	1.23-2.64
Yes	669 (69.4)	295 (30.6)	1.00			
Born in Alberta						
No	274 (68.8)	124 (31.2)	.96	.73-1.26		
Yes	395 (69.8)	171 (30.2)	1.00			
Ethnicity						
Non-Caucasian	125 (72.7)	47 (27.3)	1.12	.78-1.61		
Caucasian	702 (70.3)	296 (29.7)	1.00			
Income						
<$40,000	93 (73.8)	33 (26.2)	1.21	.79-1.85		
≥$40,000	561 (70.0)	241 (30.0)	1.00			
Residence						
Rural/other	283 (72.6)	107 (27.4)	1.16	.89-1.52		
Urban	548 (69.5)	240 (30.5)	1.00			
Personal experience						
No	315 (63.8)	179 (36.2)	.56*	.44-.72	.65	.50-.86
Yes	514 (75.8)	164 (24.2)	1.00			
Postnatal knowledge						
High	770	254	4.35*	3.04-6.22	3.88	2.65-5.67
Low	60	86	1.00			

*Met criterion for inclusion in multivariable model (p < .10).

Note. UOR = unadjusted odds ratio; AOR = adjusted odds ratio; CI = confidence interval.

### Knowledge of postnatal mental health

Over two-thirds of respondents strongly agreed/agreed that women with PPD find it difficult to respond to their baby’s cues (68.6%) with a minimal proportion indicating that they did not know (9%) (Table 2). The majority of respondents also agreed that women with PPD find it more difficult to respond to the needs of their partner or other children (79.8%). However, fewer than half of respondents reported that partners of women with PPD were more likely to experience depression (45.1%) with over 20% indicating that they strongly disagreed/disagreed (21.9%) (Table 2). In the multivariable analysis, participants with no post-secondary education and who did not know a woman with PPD were less likely to have high levels of postnatal mental health knowledge (Table 4). Those with a high level of prenatal knowledge were over three times more likely to have high knowledge of postnatal mental health.

**Table 4:** Unadjusted (UOR) and Adjusted Odds Ratios (AOR) of factors associated with knowledge of postnatal mental health in the 2012 Alberta-B survey (Alberta, Canada) (N = 1207)

**Variable**	**High level of postnatal mental health knowledge**	**Low level of postnatal mental health knowledge**	**UOR**	**95% CI**	**AOR**	**95% CI**
	**N (%)**	**N (%)**				
Sex						
Male	494 (85.2)	86 (14.8)	.67*	.47-.95	.86	.56-1.32
Female	534 (89.6)	62 (10.4)	1.00			
Employment						
Unemployed	386 (85.6)	65 (14.4)	.77	.54-1.09		
Employed	641 (88.5)	83 (11.5)	1.00			
Children (age < 18) in household						
No	671 (86.7)	103 (13.3)	.83	.57-1.20		
Yes	354 (88.7)	45 (11.3)	1.00			
Age (in childbearing years: 18–35)						
No	840 (87.5)	120 (12.5)	.93	.58-1.52		
Yes	165 (88.2)	22 (11.8)	1.00			
Marital status						
Single/widowed/divorced	312 (86.2)	50 (13.8)	.85	.59-1.23		
Married/common-law	712 (88.0)	97 (12.0)	1.00			
Education						
No post-secondary education	242 (82.6)	51 (17.4)	.58*	.40-.84	.46	.29-.73
Post-secondary	782 (89.1)	96 (10.9)	1.00			
Born in Canada						
No	175 (82.9)	36 (17.1)	.64*	.42-.96	.58	.33-1.02
Yes	853 (88.4)	112 (11.6)	1.00			
Born in Alberta						
No	351 (88.2)	47 (11.8)	.97	.65-1.44		
Yes	502 (88.5)	65 (11.5)	1.00			
Ethnicity						
Non-Caucasian	139 (81.8)	31 (18.2)	.58*	.37-.89	.62	.34-1.12
Caucasian	885 (88.6)	114 (11.4)	1.00			
Income						
<$40,000	102 (82.3)	22 (17.7)	.64*	.38-1.06	.79	.44-1.41
≥$40,000	706 (87.9)	97 (12.1)	1.00			
Residence						
Rural/Other	335 (86.1)	54 (13.9)	.84	.59-1.21		
Urban	693 (88.1)	94 (11.9)	1.00			
Personal experience						
No	406 (82.5)	86 (17.5)	.43*	.30-.62	.58	.38-.88
Yes	620 (91.6)	57 (8.4)	1.00			
Prenatal knowledge						
High	770	254	4.35*	3.04-6.22	3.47	2.28-5.29
Low	60	86	1.00			

*Met criterion for inclusion in multivariable model (p < .10).

Note. UOR = unadjusted odds ratio; AOR = adjusted odds ratio; CI = confidence interval.

### Relationship between knowledge of prenatal and postnatal mental health

Overall, more participants had high knowledge of postnatal mental health (87.4%) than prenatal mental health (70.5%) (p < .001). The Spearman’s correlation between prenatal and postnatal knowledge was small at .248 (p = .01).

## Discussion

This study provides the first Canadian data related to public views of perinatal mental health and adds to the limited body of evidence on this topic. Only one other study in Australia has described the public’s views of perinatal mental health at a population-level [13]. Overall, we found that levels of knowledge of postnatal mental health were high, while knowledge regarding prenatal mental health was significantly lower. Understanding the contributions of past mental health problems and prenatal mental health on the risk of development of depression/anxiety during and after pregnancy was also quite low. The least understood topic was the influence of prenatal mental health on child development, where only one in four participants responded accurately, and over 40% reported that they were unsure or did not know. Finally, our multivariable analyses revealed that the main determinants of both prenatal and postnatal mental health were personal experience with PPD and possessing high levels of knowledge of mental health across the perinatal period. Whereas most demographic variables were not significant, we found that older participants and those with no post-secondary education had significantly lower levels of prenatal and postnatal mental health knowledge, respectively.

Our finding that respondents were more knowledgeable about postnatal than prenatal mental health is similar to the Australian study in which 24.8% of individuals cited PPD as a major health problem, but only 7.0% of participants recognized the health implications of prenatal anxiety or depression [13]. Indeed, in this study 52% of respondents identified prenatal anxiety as ‘normal’ [13]. The limited understanding of determinants of prenatal and postnatal anxiety/depression is also consistent with this study’s report of common, erroneous beliefs regarding the causes of postnatal depression expressed by both the general public and healthcare providers [13]. The lack of knowledge about the influence of pre-conception and prenatal anxiety/depression on the risk of poor perinatal mental health [22-24] may reflect errant public belief that the primary causes of perinatal mental health problems are biological in nature –a key observation noted in the Australian study [13]. The failure to recognize the role that other factors play may underlie women’s dread regarding a diagnosis of PPD and their reticence in help-seeking in that a deterministic, biological view implies that women are bound to develop PPD and prevention is not possible.

The disparity that we observed in the public’s prenatal and postnatal mental health knowledge is notable in that qualitative studies have cited major deterrents to mental health help-seeking in pregnancy as lack of knowledge regarding symptoms, where to find help, and treatment options [5,6]. Furthermore, other studies have related low knowledge of mental health among perinatal healthcare providers to sub-optimal mental health screening behavior [18], which has been reported as less than 20% [25]. Thus, a major concern originating from our findings is that limited awareness of prenatal mental health problems may contribute to the serious under-detection and under-treatment [25,26] of prenatal mental health problems. Improving perinatal mental health knowledge (e.g., symptoms, where to seek help, treatment options) among women, their families, and healthcare providers may have a substantial impact on detection and treatment.

Emerging evidence indicates that early intervention to improve pregnant women’s mental health has enduring benefits for reducing the risk of postpartum mental health problems and poor infant health and development [27-29]. Thus, although the historical focus has been on diagnosing and treating PPD, the findings of this study highlight the need for a shift toward improving the public’s understanding of the prevalence and sequelae of prenatal anxiety and depression. The strikingly low level of understanding of the impact of prenatal mental health on child development that we found mirrors the findings of other population-based studies that highlight two key points: 1) the public dismisses the importance of the early life influences on long-term child well-being [30]; and 2) knowledge of factors that influence child development is poor [31].

The finding that older age was related to lower prenatal mental knowledge is similar to the Australian study [13] that reported that fewer older adults identified mental health problems as concerns in the postpartum period compared to younger adults. Other studies have demonstrated that low income and education are associated with low mental health literacy [32]; however, we observed that only low education was related to postnatal knowledge and neither was related to prenatal knowledge. Indeed, the most consistent predictor of prenatal and postnatal knowledge was personally knowing a woman who had experienced postpartum anxiety or depression. The positive role of personal experience on the attitudes of perinatal healthcare providers toward mental health has been reported previously [33]. Our results confirm that this is an important determinant of perinatal mental health literacy in the general public.

### Limitations

Because the questions were a component of a larger survey of diverse topics, we were limited in the scope of topics we could address and could not gather data on other important areas such as views regarding symptoms of perinatal depression/anxiety [13,19]. In addition, participants were not asked to identify their occupation, and thus we were unable to identify the views of healthcare professionals.

## Conclusions

This study highlights the lack of public awareness about prenatal mental health problems and the impact of poor perinatal mental health on future maternal mental and child development. Recommendations based on the findings include strengthening public awareness of the determinants of poor perinatal mental health and the potential influence of poor perinatal mental health on child development. However, messages regarding the association between prenatal and postnatal mental health and child outcomes need to be constructed with great care and sensitivity to avoid inflicting unnecessary guilt on childbearing women. Future research should examine the impact of perinatal mental health literacy on screening, referral, and treatment patterns.

## Abbreviations

AOR: Adjusted odds ratio; CI: Confidence interval; PRL: Population Research Laboratory; UOR: Unadjusted odds ratio.

## Competing interests

The authors declare that they have no competing interests.

## Authors’ contributions

DK conceptualized the study, participated in the development of the survey, was a consultant to the Population Research Laboratory on the testing of the survey, conducted data analysis, interpreted findings, drafted the manuscript and made final revisions. SM assisted with conceptualization of the study, data analysis, review of the findings, interpretation of the data, critical review and approval of the manuscript. M-PA reviewed and interpreted the results, critically reviewed the manuscript and approved the final version to be published. KH contributed to the development of the survey, critically reviewed the manuscript and approved the final version to be published. GL contributed to survey development, critically reviewed the manuscript and approved the final version for publication. ST aided in conceptualizing the study, participated in the development of the survey, interpreted the findings, critically reviewed the manuscript and approved the final version to be published. All authors meet criteria for authorship. All authors read and approved the final manuscript.

## Authors’ information

DK (PhD) is an Assistant Professor in the Faculty of Nursing and an Adjunct Assistant Professor in the Department of Obstetrics and Gynecology at the University of Alberta. She holds an Early Career Transition Award through the Alberta Centre for Child, Family, and Community Research. MPA (MD, FRANZCP, MB) is a perinatal psychiatrist and Professor in the Faculty of Medicine at University of New South Wales. She is also the Chair of the Perinatal and Women’s Mental Health Unit at the University of New South Wales, the Director of the St. John of God Mother-Baby Unit in Sydney, Australia, and the lead developer of the *Australian Clinical Guidelines for Perinatal Mental Health* (2011) and the International Marce Society Position Statement on *Psychosocial Assessment and Depression Screening in the Perinatal Period* (2013). KMH (PhD) is a Professor in the Faculty of Nursing and an Adjunct Professor in the Department of Psychiatry at the University of Alberta. She holds a Canada Research Chair in Stress Disorders in Women. GL (PhD) is an Associate Professor in the Faculty of Nursing at the University of Alberta and is a Certified Psychiatric Nurse. SM (PhD) is an epidemiologist with expertise in statistics, life course analysis, and mental health tool development. She is the senior scientist for the All Our Babies birth cohort study. ST is a Professor in the Department of Paediatrics and Community Health Sciences (Medicine) at the University of Calgary and an Adjunct Professor in the School of Public Health and Department of Obstetrics and Gynecology at the University of Alberta. She is a Health Scholar (Alberta Innovates-Health Solutions) and the Scientific Director of the Alberta Centre for Child, Family and Community Research.

## Pre-publication history

The pre-publication history for this paper can be accessed here:

http://www.biomedcentral.com/1471-2393/14/84/prepub

## References

[B1] Kingston D Heaman M Fell D Dzakpasu S Chalmers B Factors associated with perceived stress and stressful life events in pregnant women: findings from the Canadian maternity experiences survey Matern Child Health J 2012 16 1 158 168 10.1007/s10995-010-0732-2 21165763

[B2] Priest SR Austin MP Barnett BB Buist A A psychosocial risk assessment model (PRAM) for use with pregnant and postpartum women in primary care settings Arch women’s ment health 2008 11 5–6 307 317 18726142 10.1007/s00737-008-0028-3

[B3] Lancet Editorial: bring postnatal depression out of the shadows Lancet 2012 380 9854 1 23141599 10.1016/S0140-6736(12)61929-1

[B4] Horwitz SM Briggs-Gowan MJ Storfer-Isser A Carter AS Persistence of maternal depressive symptoms throughout the early years of childhood J women’s health 2009 18 5 637 645 10.1089/jwh.2008.1229 PMC2858294 19445615

[B5] Dennis CL Chung-Lee L Postpartum depression help-seeking barriers and maternal treatment preferences: a qualitative systematic review Birth 2006 33 4 323 331 10.1111/j.1523-536X.2006.00130.x 17150072

[B6] Chew-Graham CA Sharp D Chamberlain E Folkes L Turner KM Disclosure of symptoms of postnatal depression, the perspectives of health professionals and women: a qualitative study BMC Fam Pract 2009 10 7 10.1186/1471-2296-10-7 19159478 PMC2637839

[B7] Byatt N Moore Simas TA Lundquist RS Johnson JV Ziedonis DM Strategies for improving perinatal depression treatment in North American outpatient obstetric settings J Psychosom Obstet Gynaecol 2012 33 4 143 161 10.3109/0167482X.2012.728649 23194018

[B8] Sword W Busser D Ganann R McMillan T Swinton M Women’s care-seeking experiences after referral for postpartum depression Qual Health Res 2008 18 9 1161 1173 10.1177/1049732308321736 18689530

[B9] Flynn HA Henshaw E O’Mahen H Forman J Patient perspectives on improving the depression referral processes in obstetrics settings: a qualitative study Gen Hosp Psychiatry 2010 32 1 9 16 10.1016/j.genhosppsych.2009.07.005 20114123 PMC2818112

[B10] Byatt N Biebel K Friedman L Debordes-Jackson G Ziedonis D Pbert L Patient’s views on depression care in obstetric settings: how do they compare to the views of perinatal health care professionals? Gen Hosp Psychiatry 2013 35 6 598 604 10.1016/j.genhosppsych.2013.07.011 23969144 PMC4107904

[B11] Craig P Dieppe P Macintyre S Michie S Nazareth I Petticrew M Medical Research Council G Developing and evaluating complex interventions: the new medical research council guidance BMJ 2008 337 a1655 10.1136/bmj.a1655 18824488 PMC2769032

[B12] Gunn JM Palmer VJ Dowrick CF Herrman HE Griffiths FE Kokanovic R Blashki GA Hegarty KL Johnson CL Potiriadis M May CR Embedding effective depression care: using theory for primary care organisational and systems change Implementation sci: IS 2010 5 62 10.1186/1748-5908-5-62 PMC2925331 20687962

[B13] Highet NJ Gemmill AW Milgrom J Depression in the perinatal period: awareness, attitudes and knowledge in the Australian population Aust N Z J Psychiatry 2011 45 3 223 231 10.3109/00048674.2010.547842 21438748

[B14] Austin MP Reilly N Milgrom J Barnett B A national approach to perinatal mental health in Australia: exercising caution in the roll-out of a public health initiative Med J Aust 2010 192 2 111 20078421

[B15] Austin MP Middleton PF Highet NJ Australian mental health reform for perinatal care Med J Aust 2011 195 3 112 113 21806527 10.5694/j.1326-5377.2011.tb03236.x

[B16] Chew-Graham C Chamberlain E Turner K Folkes L Caulfield L Sharp D GPs’ and health visitors’ views on the diagnosis and management of postnatal depression: a qualitative study Br J Gen Pract 2008 58 548 169 176 10.3399/bjgp08X277212 18399021 PMC2249792

[B17] Edge D Falling through the net - black and minority ethnic women and perinatal mental healthcare: health professionals’ views Gen Hosp Psychiatry 2010 32 1 17 25 10.1016/j.genhosppsych.2009.07.007 20114124

[B18] Kim JJ La Porte LM Adams MG Gordon TE Kuendig JM Silver RK Obstetric care provider engagement in a perinatal depression screening program Arch women’s ment health 2009 12 3 167 172 10.1007/s00737-009-0057-6 19277845

[B19] Angermeyer MC Holzinger A Matschinger H Mental health literacy and attitude towards people with mental illness: a trend analysis based on population surveys in the eastern part of Germany Eur Psychiatry 2009 24 4 225 232 10.1016/j.eurpsy.2008.06.010 19361961

[B20] Schomerus G Schwahn C Holzinger A Corrigan PW Grabe HJ Carta MG Angermeyer MC Evolution of public attitudes about mental illness: a systematic review and meta-analysis Acta Psychiatr Scand 2012 125 6 440 452 10.1111/j.1600-0447.2012.01826.x 22242976

[B21] Laboratory PH 2012 Alberta Survey B Methodology Report 2012 Edmonton, Alberta: University of Alberta

[B22] Bunevicius R Kusminskas L Bunevicius A Nadisauskiene RJ Jureniene K Pop VJ Psychosocial risk factors for depression during pregnancy Acta Obstet Gynecol Scand 2009 88 5 599 605 10.1080/00016340902846049 19308810

[B23] Milgrom J Gemmill AW Bilszta JL Hayes B Barnett B Brooks J Ericksen J Ellwood D Buist A Antenatal risk factors for postnatal depression: a large prospective study J Affect Disord 2008 108 1–2 147 157 18067974 10.1016/j.jad.2007.10.014

[B24] Giardinelli L Innocenti A Benni L Stefanini MC Lino G Lunardi C Svelto V Afshar S Bovani R Castellini G Faravelli C Depression and anxiety in perinatal period: prevalence and risk factors in an Italian sample Arch of women’s ment health 2012 15 1 21 30 10.1007/s00737-011-0249-8 22205237

[B25] Mitchell AJ Coyne J Screening for postnatal depression: barriers to success BJOG 2009 116 1 11 14 10.1111/j.1471-0528.2008.01834.x 19016688

[B26] Carroll JC Reid AJ Biringer A Midmer D Glazier RH Wilson L Permaul JA Pugh P Chalmers B Seddon F Stewart DE Effectiveness of the Antenatal Psychosocial Health Assessment (ALPHA) form in detecting psychosocial concerns: a randomized controlled trial CMAJ 2005 173 3 253 259 10.1503/cmaj.1040610 16076821 PMC1180654

[B27] Milgrom J Schembri C Ericksen J Ross J Gemmill AW Towards parenthood: an antenatal intervention to reduce depression, anxiety and parenting difficulties J Affect Disord 2011 130 3 385 394 10.1016/j.jad.2010.10.045 21112641

[B28] Kozinszky Z Dudas RB Devosa I Csatordai S Toth E Szabo D Sikovanyecz J Barabas K Pal A Can a brief antepartum preventive group intervention help reduce postpartum depressive symptomatology? Psychother Psychosom 2012 81 2 98 107 10.1159/000330035 22261988

[B29] El-Mohandes AA Kiely M Gantz MG El-Khorazaty MN Very preterm birth is reduced in women receiving an integrated behavioral intervention: a randomized controlled trial Matern Child Health J 2011 15 1 19 28 10.1007/s10995-009-0557-z 20082130 PMC2988881

[B30] Davey L Talking child mental health and the core story of child development in Alberta 2010 Washington, D.C: FrameWorks Institute

[B31] Rikhy S Tough S Trute B Benzies K Kehler H Johnston DW Gauging knowledge of developmental milestones among Albertan adults: a cross-sectional survey BMC public health 2010 10 1 183 10.1186/1471-2458-10-183 20377910 PMC2859399

[B32] Reavley NJ Jorm AF Recognition of mental disorders and beliefs about treatment and outcome: findings from an Australian national survey of mental health literacy and stigma Aust N Z J Psychiatry 2011 45 11 947 956 10.3109/00048674.2011.621060 21995330

[B33] Leddy M Haaga D Gray J Schulkin J Postpartum mental health screening and diagnosis by obstetrician-gynecologists J Psychosom Obstet Gynaecol 2011 32 1 27 34 10.3109/0167482X.2010.547639 21261561

